# Assessing the biomarker potential of plasma ITPKB expression levels in Alzheimer's Disease

**DOI:** 10.1002/alz70856_105437

**Published:** 2026-01-10

**Authors:** Zeynep Dilhan Ün, Nagehan Ersoy Tunali

**Affiliations:** ^1^ İstanbul Medeniyet University, İstanbul, İstanbul, Turkey; ^2^ Science and Advanced Technologies Research Center (BİLTAM), İstanbul, İstanbul, Turkey; ^3^ Science and Advanced Technologies Research Center (BİLTAM), İstanbul, İstanbul, Turkey; ^4^ Istanbul Medeniyet University, İstanbul, İstanbul, Turkey

## Abstract

**Background:**

Neuroinflammation has garnered attention in Alzheimer's Disease (AD) due to its exacerbating effects on disease pathogenesis. Dysregulation of ITPKB (inositol‐trisphosphate 3‐kinase B) was shown to contribute to disease pathogenesis via neuroinflammation and tau phosphorylation in *in vitro* and *in vivo studies*, which suggests its potential as a biomarker for AD. In this study, we aimed to investigate the expression levels of ITPKB in the plasma samples of AD patients and healthy controls in order to assess its biomarker potential for the first time in the literature.

**Method:**

We collected blood samples from AD patients at different stages and healthy controls. The subjects were clinically categorized based on neurological examination and MMSE test scores. Following plasma isolation, total RNA was extracted and ITPKB mRNA expression levels were quantified using the qRT‐PCR method. The results were analyzed by the ddCt method, with β‐actin serving as the housekeeping gene for normalization. *p*‐values < 0.05 were considered statistically significant. Pearson's r correlation test was performed to assess the relationship between ITPKB expression levels and MMSE scores across all samples.

**Result:**

ITPKB gene expression was found to be significantly downregulated in mild to moderate and moderate AD patients compared to controls. Although ITPKB expression tends to be lower in mild AD subjects, this difference was not statistically significant. A significant downregulation trend was found between mild to moderate and moderate AD patients as well. Additionally, a statistically significant positive correlation was determined between ITPKB expression levels and MMSE scores (*r* = 0.68).

**Conclusion:**

Current literature points out that ITPKB contributes to AD pathogenesis, particularly through neuroinflammation and tau phosphorylation. Based on this background, we hypothesized that ITPKB could serve as a diagnostic biomarker for AD. However, our preliminary results indicate that ITPKB is downregulated in AD plasma samples compared to controls across all disease stages. Moreover, ITPKB expression levels showed a positive correlation with MMSE scores, suggesting a potential alternative role in AD progression. Once these results are validated with further studies and underlying mechanisms are identified, ITPKB expression can be regarded as a potential plasma biomarker in AD.

